# Editorial

**Published:** 1841-07-10

**Authors:** 


THE MEDICAL EXAMINER.

PHILADELPHIA, JULY 10, 1841.

### MEDICAL REFORM IN GREAT BRITAIN AND THE UNITED STATES.

The subject of Medical Reform, after long agitation in Great Britain, has been recently brought before Parliament. Bills on the subject have been introduced during the present session into the House of Commons; but, owing to the premature dissolution of that body about to take place, they will hardly be acted upon until the meeting of the new Parliament. The essential character of the proposed reform consists in the abolition of the special privileges of the various corporations which now conduct the business of medical education in Great Britain. In the place of these, it is proposed to establish a uniform system of examinations for degrees or licenses to practice, under the superintendance of a representative body, to be periodically chosen by the Profession at large. The details of the plan by which these principles are to be carried out, are, in general, of mere local importance, and not worth the particular attention of the American reader; but the subject in its main features contains points of consideration of parallel interest in our own country. These proposed bills for medical reform encounter considerable opposition. While the evils of the present system are generally admitted, plausible objections are urged to the plan by which they are to be remedied. On the one hand, all attempts to destroy the influence and authority of the existing medical colleges and corporations, are resisted by those directly and indirectly interested in these "bulwarks of protection to the honour, security, and legitimate interests of their members." While on the other hand, objection is taken to the tendency of periodical elections of a general representative body by the profession at large, to produce and keep alive among them, agitation and dissension, and to divert their attention from the calm prosecution of their more serious duties. Still, it is conceded, even by the most conservative, and to this concession we ask the special attention of our readers, that the existence of a multiplicity of rival avenues to the privileges of practice, exercises the most prejudicial effect upon the interests of the profession, and upon the progress of medical science. The most tenacious advocates of the existing medical colleges, admit the serious objections to the exercise by each, *independently of the rest,* of the privilege of conferring licences and degrees, while the only point at issue seems to be, whether this privilege shall be exercised by the colleges in a mass through their delegates,—by the great body of the profession through their own representatives,—or, as a plan less liable to produce convulsions and disputes, by a board, nominated by government. Here then, we find the profession of Great Britain and Ireland bearing nearly unanimous testimony to the evils arising from the existing mode of conferring the privilege to practice, and calling out for a change. The attention of Parliament and of public opinion is carefully directed to the subject, and a due settlement of it is delayed, only by the sudden development of an unusual political crisis.

The same bad system of qualifying practitioners of medicine, every one is aware, prevails in this country, as in Britain. It is a system, the ill effects of which are so palpable, as hardly to require illustration. If the possession of a diploma be a necessary guarantee to the public on the part of the practitioner of medicine, to protect it from incompetency, the authority to grant this important document should surely not be entrusted to rival corporations dependent for reputation and emolument upon the number of diplomas they may grant. The obvious tendency of the existing system, is to degrade the standard of qualification for admission into the profession. The personal character of the members of the various corporations is no security that they will exercise their privilege with due discrimination. General and sad experience proves that every where, individual responsibility is lost in the discharge of corporate duties. Like "merchants," it may be said,

"*Physicians*, men immaculate, perhaps,
In all their private functions, when combin'd
In corporations, seem at once to lose
Their nature."

At the recent examination in this city of candidates for the post of assistant surgeon in the navy, about two-thirds, graduates in medicine, were rejected as incompetent. A fact like this is an unanswerable argument for a radical change in the mode of examinations for degrees in medicine. A uniform system, by a board of able examiners, not interested in the number of licences to be conferred, it cannot be disputed, should be substituted for the present fluctuating and flimsy style of examinations. We do not think that the real interests of existing institutions will be impaired by the sur render of the power to confer degrees, while the great good of the profession will be vastly promoted by it. We shall pursue this subject through several articles, and continue to agitate it while the evil in question remains unremedied.

---

ELISHA BARTLETT, M.D., has been appointed Professor of the Theory and Practice of Physic, in Transylvania University—a selection likely to promote the interest of the school.

